# A validated test has been developed for assessment of manual small incision cataract surgery skills using virtual reality simulation

**DOI:** 10.1038/s41598-023-32845-5

**Published:** 2023-06-30

**Authors:** Daniel E. Hutter, Line Wingsted, Sanja Cejvanovic, Mads Forslund Jacobsen, Luis Ochoa, Karla Pamela González Daher, Morten la Cour, Lars Konge, Ann Sofia Skou Thomsen

**Affiliations:** 1 Copenhagen Academy for Medical Education and Simulation (CAMES), Copenhagen, Denmark; 2 Department of Ophthalmology, Rigshospitalet, Glostrup, Denmark; 3 Instituto Mexicano De Oftalmología (IMO), Querétaro, Mexico

**Keywords:** Surgery, Lens diseases

## Abstract

This study investigates the validity evidence of metrics used for the assessment of surgical skills for Manual Small Incision Cataract Surgery (MSICS) in a virtual reality simulator. MSICS surgery is a low-cost, low-technology cataract surgery technique, which is widely used in low- and middle-income countries. However, there is a lack of cataract surgeons globally, and efficient and evidence-based training of new surgeons is needed. In order to investigate the validity of simulator metrics, we included three groups of participants: (1) MSICS novices who were ophthalmologists with no cataract surgery experience, (2) MSICS novices who were experienced phacoemulsification cataract surgeons, but with no MSICS experience, and (3) experienced phacoemulsification and MSICS surgeons. The evaluation included 11 steps of the MSICS procedure, and all simulator metrics for those steps were reviewed. Of the 55 initial metrics, 30 showed high positive discriminative ability. A test passing score of 20 out of 30 was established, and one of 15 novices with no MSICS experience (mean score 15.5) and 7 out of 10 experienced MSICS surgeons (mean score 22.7) passed the test. We have developed and established validity evidence for a test for MSICS skills in a virtual reality simulator for future use in proficiency-based training and evidence-based testing of training interventions.

## Introduction

Cataract blindness affects twenty million people worldwide, primarily in low- and middle-income countries^1^. This blindness is reversible with successful cataract surgery. Manual Small Incision Cataract Surgery (MSICS) is a low-cost, low-technology cataract surgery technique that has been found to have outcomes comparable to the more expensive, high-technology technique of phacoemulsification^2^. However, the shortage of eye surgeons globally has led to a backlog of patients in need of cataract surgery^3^. Additionally, training new surgeons is challenging due to the increased risk of complications in cataract surgery performed by trainees.

Simulation-based training (SBT) has been shown to improve the operating room performance of surgeons, resulting in better patient outcomes^4–8^. While previous research has focused on the use of simulators for phacoemulsification cataract surgery, the use of SBT for training in MSICS could also be beneficial. This approach may be an effective way to train more surgeons in MSICS. A non-profit humanitarian organization called HelpMeSee has developed a virtual reality simulator with haptic feedback to train surgeons in the safe performance of MSICS. The aim of HelpMeSee is to increase the number of MSICS surgeons operating globally, increase the number of MSICS surgeries performed, and restore vision to a greater number of individuals worldwide (https://helpmesee.org).

Mastery learning is an approach to SBT whereby trainees are assessed at baseline and during training, with an end goal of attaining a minimum passing standard^9^. To ensure evidence-based proficiency assessment in mastery learning, it is necessary to demonstrate evidence of validity for the assessment tools used^10^. According to STANDARDS for Educational and Psychological Testing^11^, there are five sources of validity evidence. Demonstrating evidence in these five areas supports an argument that a test has validity in a particular context. The sources of validity evidence are based on test content, response processes, internal structure, relations to other variables, and consequences of testing.

Currently, there is no objective, virtual reality simulation assessment of performance in the steps of MSICS surgery with established validity evidence. In order to address this issue, our aim was to develop a test of MSICS competencies using the HelpMeSee simulator and to provide validity evidence for use of the automatic and unbiased outcome metrics.

## Methods

The study was conducted as a prospective study. It was carried out at the Copenhagen Academy for Medical Education and Simulation (CAMES), Capital Region of Denmark, and Instituto Mexicano De Oftalmología (IMO), Querétaro, Mexico from October 2020 to April 2021.

This study adhered to the tenets of the Declaration of Helsinki. No human patients were involved in the study. The Ethics Committee of the Capital Region of Denmark ruled that approval was not required for this study (protocol no. 20051000).

The HelpMeSee simulator for MSICS was used for this study. A team of MSICS clinical experts with experience in simulator development included 11 steps in the HelpMeSee MSICS Standard Procedure for testing. Careful consideration was given to selecting clinically relevant assessment parameters for the related outcome metrics. To ensure the best possible validity evidence for *test content*, we decided to include all 11 steps in our test.

Three groups of participants were defined: (1) ophthalmologists with no cataract surgery experience, (2) experienced phacoemulsification cataract surgeons with no MSICS experience, and (3) surgeons experienced in both phacoemulsification and MSICS cataract surgery. Inclusion criteria for each of the groups were: (1) ophthalmologists employed at an ophthalmology department without any cataract surgery experience, (2) surgeons with > 1000 phacoemulsification procedures and no MSICS experience, and (3) surgeons with > 1000 phacoemulsification procedures and > 500 MSICS operations. All novices were recruited from the Department of Ophthalmology, Rigshospitalet, Denmark and IMO, Mexico. Cataract surgeons were recruited from ophthalmology departments or private specialist clinics in the Zealand region of Denmark and from IMO. All surgeons had to be active surgeons at the time of the study, having operated within the past month. Exclusion criteria for all three groups included previous experience with the HelpMeSee simulator for more than 2 h during the past 3 months. In addition, participants who had more than 2 h of experience on another virtual reality simulator in the past 3 months were excluded.

### Study size

In order to assume normal distribution of test scores, we aimed for at least 10 experienced MSICS surgeons and 10 novices in MSICS^12^.

### Data collection

The data collection was standardized to avoid threats to validity by ensuring that the administrator of the test did not influence the process and that every participant had a fair and equal test administration (validity evidence towards *response process*). During testing, three authors (SC, LW, LO) gave verbal instructions to all participants based on a written document to ensure that the same instructions were given to all participants. Only the technical aspects of their performance were intended to be assessed.

### Overview of the standardized data collection procedure for each participant

Each participant was assigned a unique ID number and was provided with information about HelpMeSee and the study. Informed consent was obtained and a questionnaire collecting demographic data and experience was completed. Stereoacuity using the TNO test (Laméris Ootech BV, 19th edition) was measured. Each participant viewed an approximately 12-min video of the HelpMeSee MSICS Standard Procedure (https://vimeo.com/426195663). Warmup lasted for 10 min, using the first two assignments. Practical information was provided, including instructions on how to start the simulation, the objectives for each task, and descriptions of the instruments, the training tools, and the scoring parameters. For the data collection, the entire procedure was required to be completed two times, with the possibility of a short break between each procedure. The completion of both procedures could not exceed 2 h. To reduce the risk of fatigue, if the time exceeded this limit, the participant would need to return later. The participants were instructed to complete each task, and they were allowed to determine when each task was successfully finished or ended due to a complication. During the attempts, the participants were not able to view the simulator metrics.

In order to evaluate performance, 55 different metrics were assessed across all the steps of the procedure. Some of the metrics recorded whether an attempt resulted in an outcome within a designated range, such as the length of the inner tunnel limit during scleral tunnel dissection. Other metrics recorded whether a specific complication occurred, such as uveal prolapse during creation of a scleral groove.

### Statistical analysis

The HelpMeSee simulator uses proprietary scoring logic that was converted to a binary output, with a score of 1 indicating a pass and a score of 0 indicating a fail for each metric for each task. Imputation using the grand mean was utilized to fill in 64 missing values out of a total of 1275 in the data set^13^. The data was then imported into SPSS software version 26.0 (SPSS, Inc., Chicago, IL) for statistical analysis.

Item-Total statistics were used to reduce the number of metrics used in the test due to either low discrimination or nondiscrimination of individual metrics based on Corrected Item-Total Correlation^14^. All items with negative discrimination were also removed. Inter-metric reliability analysis was performed using intraclass correlation coefficient to calculate Cronbach’s Alpha and associated confidence intervals.

Mean test scores for novice MSICS surgeons (including both the non-surgeons and the phacoemulsification-only cataract surgeons) and experienced MSICS surgeons were compared using Independent Samples T-tests.

The Contrasting Groups’ Method was employed to establish a proficiency level^15^. The pass/fail score was determined at the intersection between the distributions of test scores obtained from novices and experienced MSICS surgeons.

## Results

The MSICS novice group included 15 participants, 10 of whom were ophthalmologists with no experience in phacoemulsification or MISCS and 5 of whom were experienced phacoemulsification cataract surgeons without MSICS experience. The experienced MSICS group consisted of 10 surgeons who were experienced in both phacoemulsification and MSICS cataract surgery.

Table 1 shows the original 55 metrics recorded, and the 30 metrics included in the test.

**Table 1:** Metrics included and excluded from the evidence-based test to assess MSICS performance.

Task	Included items	Excluded items	Nov %	Exp %	Item disc
*Creating a scleral groove*	*Appropriate length of the groove*		47	50	0.34
		Avoiding uveal prolapse			0.07
*Dissecting a tunnel*	*Appropriate length of the inner tunnel limit*		7	100	0.27
	*Avoiding premature entry*		87	100	0.47
		Avoiding laceration of the outer wall			0.00
		Appropriate length of the tunnel			− 0.18
		Avoiding perforation of the outer wall (buttonholes)			− 0.10
		Avoiding uveal prolapse			0.00
*Making a paracentesis and injecting viscoelastic*	*Avoiding contact with the iris during paracentesis*		93	90	0.13
	*Appropriate size of the paracentesis*		40	50	0.20
	*Avoiding contact with the iris during viscoelastic injection*		47	59	0.40
		Avoiding endothelial touch during paracentesis			− 0.58
		Avoiding endothelial touch during viscoelastic injection			− 0.22
*Entering the anterior chamber with keratome*	*Avoiding contact with the iris*		60	100	0.31
	*Avoiding lateral tunnel laceration*		93	100	0.35
	*Appropriate length of the inner tunnel opening*		0	30	0.46
	*Avoiding premature entry*		87	100	0.24
		Avoiding buttonhole			0.00
		Avoiding endothelial touch			− 0.23
*Making a Capsulorrhexis*	*Appropriate maximum size of capsulorrhexis*		7	50	0.69
	*Appropriate minimum size of capsulorrhexis*		13	80	0.67
	*Avoiding runout*		93	100	0.28
	*Avoiding zonular breakage*		7	70	0.64
	*Avoiding zonular breakage more than 50%*		60	100	0.26
		Avoiding contact with the iris			− 0.29
		Avoiding endothelial touch			0.01
*Performing hydrodissection and nucleus dislocation*	*Achieving complete hydrodissection*		93	100	0.28
	*Avoiding endothelial touch during nucleus dislocation*		69	60	0.11
	*Avoiding zonular breakage during hydrodissection*		93	100	0.21
	*Avoiding zonular breakage during nuclear dislocation*		56	80	0.23
	*Avoiding zonular breakage more than 50% during nuclear dislocation*		96	100	0.16
		Avoiding contact with the iris during hydrodissection			0.00
		Avoiding contact with the iris during nucleus dislocation			0.06
		Avoiding cortical hydration			0.02
		Avoiding endothelial touch during hydrodissection			0.11
		Avoiding partial hydrodissection			0.00
		Avoiding zonular breakage more than 50% during hydrodissection			0.00
*Delivering the nucleus*	*Avoiding endothelial touch*		73	100	0.49
	*Avoiding iris pull*		73	100	0.06
*Removing the cortex*	*Amount of residual cortex (0%)*		0	60	0.73
	*Avoiding capsular aspiration*		7	20	0.18
	*Avoiding iris aspiration*		87	100	0.13
	*Location of residual cortex*		0	60	0.73
	*Avoiding zonular breakage*		0	40	0.57
	*Avoiding zonular breakage more than 50%*		0	60	0.73
		Avoiding endothelial touch			− 0.15
		Avoiding posterior capsular rupture			0.00
*Inserting the IOL*	*Avoiding lost IOL*		80	100	0.10
	*Avoiding zonular breakage more than 50%*		87	100	0.27
Dialing the IOL		Avoiding endothelial touch			0
		Achieving IOL completely inside the capsular bag			0
		Avoiding zonular breakage			0
		Avoiding zonular breakage more than 50%			0
Hydrating the paracentesis site		Avoiding contact with the iris			− 0.14
		Avoiding endothelial touch			− 0.14

Significant values are in italics.

*Nov* novices, *Exp* experienced, *Item disc* item discrimination.

Intraclass correlation coefficient was calculated with a Cronbach’s Alpha of 0.86 with a 95% confidence interval lower bound of 0.77 and upper bound of 0.93.

Using the 30-item test, the novices had a mean score of 15.5 (SD 3.0) and the experienced had a mean score of 22.7 (SD 4.3), p < 0.001.

**Figure 1:**
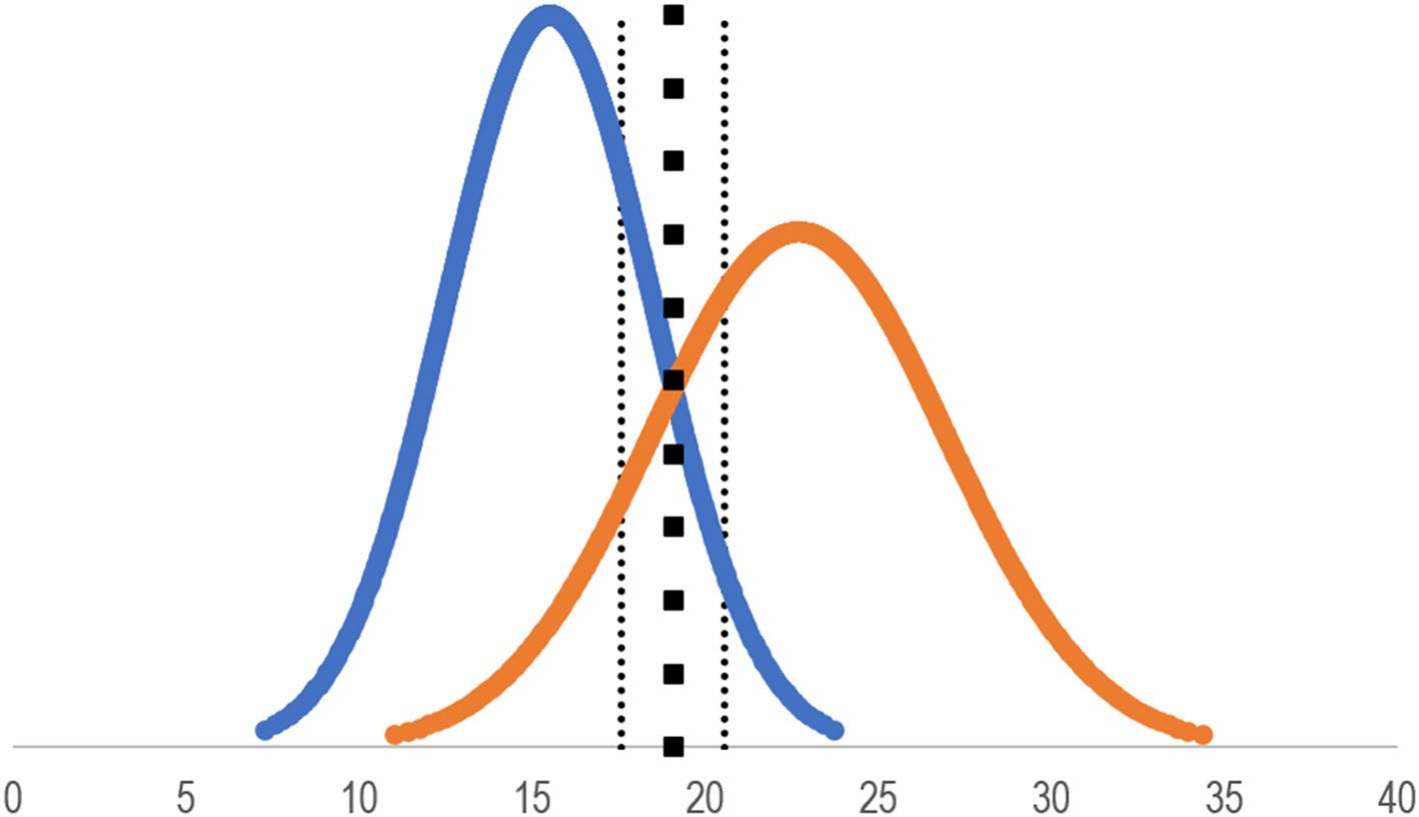
The establishment of a credible pass/fail standard using the contrasting groups’ method. The curves are constructed based on the mean scores and standard deviations of novice (blue) and experienced (orange) operators, respectively. The big box line at the intersection marks the pass/fail standard of 20 points and the small, dotted lines show the 95% confidence intervals of the standard.

Figure 1 shows that the pass/fail standard was established at 20 points (out of 30). This resulted in only 1 out of 15 novices passing the test (6.7% false positives) and 3 out of 10 experienced surgeons failing the test (30% false negatives).

## Discussion

We examined the validity of the original 55 metrics captured by the HelpMeSee simulator for the complete MSICS standard procedure and developed an evidence-based test that was narrowed down to 30 metrics. A passing score on this test was determined to be 20 out of 30. Only 1 of the 15 novice MSICS surgeons (mean score 15.5) obtained a passing score, while 7 out of 10 experienced MSICS surgeons (mean score 22.7) passed the test.

In the development of an evidence-based assessment tool, we used only metrics provided by the simulator. These metrics were based on whether the study participant's performance of a step of the procedure resulted in a particular measurement which was within a specified range or avoided a specific error during that step. Using simulator-provided metrics has several benefits, including ease of access and the ability to make numerical comparisons between test takers. Utilizing metrics directly from the simulator also eliminates bias which may occur when performance is evaluated by human raters^16^. This is the first study to develop an evidence-based test for the assessment of MSICS performance using the HelpMeSee simulator, but a similar test based solely on simulator metrics was created for phacoemulsification cataract surgery using the EyeSi virtual reality simulator^8^. That test ended up using 7 out of 13 modules (54%) that demonstrated significant discriminative ability, which is similar to the discriminative ability of the metrics used in our test, which included 30 out of 55 (55%).

While using only simulator metrics has some advantages, there are also some drawbacks. By using only binary test scores, some of the richness of information that can be obtained through the use of global rating scales is lost^17^. Trained raters can evaluate surgical performance by live viewing or video review allowing for a more comprehensive evaluation, but this approach is time consuming, resource intensive, and prone to bias^18,19^. A color graphics overlay captures not only length and width of the scleral-corneal dissection, but also variations in the depth and deviations in the shape from recommended guidelines. However, there is currently no automated way to assess performance using such a graphics overlay. A way to improve assessment might be to use artificial intelligence to analyze the graphics overlays provided by the simulator.

It is also important to note that, since our validated test only uses those metrics captured by the HelpMeSee simulator for assessment, it is limited by the metrics that are available and may not adequately represent all aspects of performance. Construct underrepresentation is a major threat to validity^20^. In addition, the number of metrics captured for each step is fixed by the simulator. For example, the step “Delivering the Nucleus” has only two associated metrics, while the step “Performing Hydrodissection and Nucleus Dislocation” has 11 metrics. The balance of our test is thus impacted by the weight given to the different steps. It is possible that the test could be improved by considering additional metrics or adjusting the weighting of steps. As new versions of the HelpMeSee simulator software are released, updates can include better descriptions of the metrics to remove ambiguity and clarify exactly what is being measured during specified simulator tasks. Also, combining simulator metrics with expert raters might be helpful, especially during final assessment.

Interestingly, none of the 5 experienced phacoemulsification cataract surgeons, who were not experienced MSICS surgeons, passed the test. Despite the similarities in some of the steps in MSICS and phacoemulsification, this finding suggests that experience in performing phacoemulsification does not necessarily translate to proficiency in MSICS. This agrees with previous studies which have shown that microsurgical motor learning is highly task specific^21–24^. Thomsen’s study compared the performance of ophthalmology residents who had received training in cataract surgery to those who had not, and found no significant difference in their ability to perform vitreoretinal procedures on the EyeSi simulator. It would be unlikely for a cataract surgeon to begin performing vitreoretinal surgery without first obtaining additional fellowship training, but it would be much more likely that a phacoemulsification surgeon might start performing MSICS without additional formal training. Therefore, we may underestimate the additional knowledge and skills required to learn MSICS because it is not intuitive that ocular surgical skills in one procedure do not necessarily transfer to another ocular procedure.

A strength of our study design was the use of a standardized method for collecting data. This standardized method allowed for collecting data internationally from multiple centers and ensured that all the steps of the MSICS procedure were included in the development of our test to capture the complexity of the procedure.

This study has some limitations. One limitation is the small number of participants, which is common in medical education research^12^. Another limitation is that the simulator and its associated metrics are designed to assess performance based on the technique used by HelpMeSee for its MSICS Standard Procedure. Many experienced MSICS surgeons may use techniques that differ from the HelpMeSee Standard Procedure, and thus they may have encountered difficulties successfully completing a particular task during data collection that they may not have encountered during live surgery. Lastly, we used the number of cataract surgeries performed and the date when they were last performed as a proxy for competence in MSICS. However, we did not capture the frequency with which the experienced surgeons had specifically performed MSICS, which could potentially impact their level of competence in this procedure.

Several studies have shown the effectiveness of virtual reality simulators in training and assessing phacoemulsification cataract surgery skills^7,25^. With our establishment of an evidence-based test, it is now possible to provide proficiency-based training using the HelpMeSee simulator for MSICS. While we found no inter-procedural transfer of skills from phacoemulsification to MSICS, phacoemulsification surgeons may still require reduced training time due to their previous knowledge and experience. Using our test, simulation-based training can be customized to match the background of the surgeon. The proficiency of the surgeon can then be assessed before progressing on to supervised live surgery using simulator metrics which have been shown to have validity evidence. In the future, this evidence-based test for MSICS surgery can be used to investigate the effectiveness of various training interventions or to design curricula for MSICS training.

## Conclusion

With this study, we have demonstrated the development of an evidence-based test for MSICS surgery using virtual reality simulation. This test can be used for assessment during MSICS training. In addition, it can be used as a test when evaluating different training methods for teaching MSICS.

## Data Availability

The datasets generated during and/or analyzed during the current study are available from the corresponding author on reasonable request.

## References

[1] Bourne RRA (2017). Magnitude, temporal trends, and projections of the global prevalence of blindness and distance and near vision impairment: A systematic review and meta-analysis. Lancet Global Health.

[2] Dean WH (2019). Ophthalmic simulated surgical competency assessment rubric for manual small-incision cataract surgery. J. Cataract Refract. Surg..

[3] Yorston D (2005). High-volume surgery in developing countries. Eye.

[4] Adnane I, Chahbi M, Elbelhadji M (2020). Virtual simulation for learning cataract surgery. J. Fr. Ophtalmol..

[5] Ferris JD (2020). Royal College of Ophthalmologists’ National Ophthalmology Database study of cataract surgery: Report 6. The impact of EyeSi virtual reality training on complications rates of cataract surgery performed by first and second year trainees. Br. J. Ophthalmol..

[6] McCannel CAMD, Reed DCMD, Goldman DRMD (2013). Ophthalmic surgery simulator training improves resident performance of capsulorhexis in the operating room. Ophthalmology.

[7] Staropoli PC (2018). Surgical simulation training reduces intraoperative cataract surgery complications among residents. Simul. Healthc..

[8] Thomsen ASS, Kiilgaard JF, Kjærbo H, la Cour M, Konge L (2015). Simulation-based certification for cataract surgery. Acta Ophthalmol..

[9] McGaghie WC, Wayne DB, Barsuk JH, Issenberg SB (2021). Deliberate practice and mastery learning contributions to medical education and improved healthcare. J. Expert..

[10] Grantcharov TP, Reznick RK (2008). Teaching procedural skills. BMJ.

[11] American Educational Research Association (2014). Standards for Educational and Psychological Testing.

[12] Bloch R, Norman G (2012). Generalizability theory for the perplexed: A practical introduction and guide: AMEE Guide No. 68. Med. Teach..

[13] Beland S, Pichette F, Jolani S (2016). Impact on Cronbach’s alpha of simple treatment methods for missing data. Tutorials Quant. Methods Psychol..

[14] Downing, S. M., Juul, D. & Park, Y. S. in *Assessment in health professions education* (eds Rachel Yudkowsky, Yoon Soo Park, & Steven M Downing) Ch. 5, (Routledge, 2019).

[15] Jørgensen M, Konge L, Subhi Y (2018). Contrasting groups' standard setting for consequences analysis in validity studies: Reporting considerations. Adv. Simul..

[16] Devine, L. A., McGaghie, W. C. & Issenberg, S. B. in *Assessment in health professions education* (eds Rachel Yudkowsky, Yoon Soo Park, & Steven M Downing) Ch. 14, (Routledge, 2019).

[17] Ilgen JS, Ma IWY, Hatala R, Cook DA (2015). A systematic review of validity evidence for checklists versus global rating scales in simulation-based assessment. Med. Educ..

[18] Konge L (2012). Reliable and valid assessment of clinical bronchoscopy performance. Respiration.

[19] la Cour M, Thomsen ASS, Alberti M, Konge L (2019). Simulators in the training of surgeons: Is it worth the investment in money and time? 2018 Jules Gonin lecture of the Retina Research Foundation. Graefes Arch. Clin. Exp. Ophthalmol..

[20] Downing SM (2002). Threats to the validity of locally developed multiple-choice tests in medical education: Construct-irrelevant variance and construct underrepresentation. Adv. Health Sci. Educ. Theory Pract..

[21] Bjerrum FMD (2015). Procedure-to-procedure transfer in laparoscopic simulator training: Results from a randomized trial. J. Am. Coll. Surg..

[22] Petersen SB (2022). Pretraining of basic skills on a virtual reality vitreoretinal simulator: A waste of time. Acta Ophthalmol..

[23] Selvander M, Åsman P (2012). Virtual reality cataract surgery training: Learning curves and concurrent validity. Acta Ophthalmol..

[24] Thomsen ASS, Kiilgaard JF, Cour M, Brydges R, Konge L (2017). Is there inter-procedural transfer of skills in intraocular surgery? A randomized controlled trial. Acta Ophthalmol..

[25] Thomsen AS (2017). Operating room performance improves after proficiency-based virtual reality cataract surgery training. Ophthalmology.

